# Gastric squamous metaplasia observed by image‐enhanced endoscopy

**DOI:** 10.1002/deo2.219

**Published:** 2023-03-14

**Authors:** Eri Iwata, Mitsushige Sugimoto, Yoshika Akimoto, Mariko Hamada, Ryota Niikura, Naoyoshi Nagata, Takao Itoi, Takashi Kawai

**Affiliations:** ^1^ Department of Gastroenterological Endoscopy Tokyo Medical University Hospital Tokyo Japan; ^2^ Department of Gastroenterology and Hepatology Tokyo Medical University Hospital Tokyo Japan

**Keywords:** ectopic mucosa, gastric squamous metaplasia, image‐enhanced endoscopy, narrow‐band imaging, texture and color enhancement imaging

## Abstract

A 61‐year‐old *Helicobacter pylori*‐positive female with gastroesophageal reflux disease has undergone surveillance endoscopy every year for 13 years at Tokyo Medical University Hospital. At the first surveillance in 2009, conventional white light endoscopy showed a 10‐mm whitish slightly depressed lesion at the lesser curvature of the gastric cardia. This lesion gradually increased in size to 15 mm over the 13‐year observational period. Indigo carmine chromoendoscopy, narrow band imaging, and texture and color enhancement imaging in both mode 1 and mode 2 clearly emphasized the presence of a depressed whitish mucosa around the gastric mucosa compared with white light imaging. None of the three image‐enhanced endoscopy techniques showed any abnormality in the vascular or structural pattern. Pathological findings showed squamous epithelium without atypia and revealed no evidence of malignancy in the stomach. We thus report a case of gastric squamous metaplasia without gastric neoplastic lesion in the gastric cardia whose lesions were endoscopically observed to change the size for more than 10 years and whose lesions were endoscopically evaluated with a texture and color enhancement imaging mode 1 and mode 2 and narrow band imaging.

## INTRODUCTION

Metaplasia, including Barrett's esophagus in the esophagus and ectopic gastric mucosa in the esophagus and duodenum, is often observed in gastrointestinal endoscopy during routine health checks. Metaplasia generally involves the histological transformation of one differentiated cell type to another. For example, in Barrett's esophagus, esophageal squamous cell epithelium at the esophagogastric junction transforms into the columnar epithelium. Gastric squamous metaplasia is a relatively rare type of metaplasia observed in the stomach (Table 1).^1^, ^2^, ^3^, ^4^ The endoscopic appearance of gastric squamous metaplasia is whitish depressed lesions mainly located in the gastric cardia and antrum.^1^, ^2^, ^3^, ^4^ The etiology of gastric squamous metaplasia remains unclear, and it is important to differentially diagnose it from neoplastic lesions and ulcerative diseases.

**TABLE 1:** Cases of gastric squamous metaplasia without neoplastic lesions who reported in English since 1985.

Author (year)	Age (sex)	Size (mm)	Location (U/M/L)	Observation period (years)	Changes	White light imaging	NBI/magnified NBI	TXI	Esophageal disease	Gastric disease	Systemic disease
Tolia V (1986)	13 (M)	NA	L	NA	NA	The zone of pale mucosa	NA	NA	NA	Atrophic gastritis	Ulcerative colitis
Takeda (2000)	60 (F)	40	U	1	No change	Slightly depressed, and several reddish spots inside	NA	NA	NA	Gastric ulcer	Na
Takeda (2000)	65 (F)	40	U	1	No change	Irregular whitish mucosal lesion	NA	NA	NA	Gastric ulcer	Na
Cho (2008)	81 (M)	8	L	2	No change	Linear depressed whitish lesion	NA	NA	NA	Na	Pulmonary tuberculosis
Fujisawa (2009)	70 (F)	40	U	5	Increase of size	White depressed lesion	IPCL in white depressed mucosa	NA	GERD, SSBE hiatus hernia	Atrophic gastritis	Liver cirrhosis
Jeon (2013)	79 (M)	NA	U	3	NA	Whitish flat lesion	NA	NA	NA	Gastric cancer	Na
Ishida (2015)	89 (M)	15	L	NA	NA	Whitish depressed lesion	IPCL‐like vascular structures	NA	NA	Na	Anemia
Oide (2018)	90 (M)	NA	U	NA	NA	Whitish depressed lesion, demarcated	NA	NA	NA	Gastric cancer	Na
Iwatsubo (2019)	80 (M)	8	L	6	No change	Whitish depressed lesion	IPCL vessel in the depressed area	NA	NA	Gastric cancer	Na
de Miranda Neto (2000)	34 (M)	NA	M/L	NA	NA	Gray‐white to whitish mucosal lesion	NA	NA	GERD	Na	Vitamin B_12_ deficiency
Iwamuro (2021)	45 (M)	NA	M/L	9	NA	Whitish depressed lesion	Whitish lesions. IPCL was not observed	NA	NA	Atrophic gastritis	Follicular lymphoma
Our case	74 (F)	15	U	13	Increase of size	Whitish depressed lesion	Depressed whitish lesions	Depressed whitish lesions and surrounding reddish gastric mucosa	GERD, SSBE hiatus hernia	Gastric cancer and adenoma	NA

Abbreviations: F, female; GERD, gastroesophageal reflux disease; IPCL, intrapapillary capillary loop; L, lower third of stomach; M, male; M, middle third of stomach; NA, not available; NBI, narrow‐band‐imaging; SSBE, short segment Barrett's esophagus; TXI, texture and color enhancement imaging; U, upper third of stomach.

Recent advances in image‐enhanced endoscopy (IEE), including narrow‐band imaging (NBI) and texture and color enhancement imaging (TXI), have improved the detection rate of gastric cancer and intestinal metaplasia,^5^ esophageal adenocarcinoma^6^ and Barrett's epithelium. However, reports on endoscopic detection and evaluation of gastric squamous metaplasia using IEE are limited.

Here, we report a case of gastric squamous metaplasia that increased in size over a follow‐up period of more than 10 years and was evaluated using the new optical IEE modalities TXI and NBI.

## CASE REPORT

A 74‐year‐old female with gastroesophageal reflux disease grade M and the reflux‐related symptom was given esomeprazole 20 mg for 13 years at Tokyo Medical University Hospital. During the 13‐year observational period, the patient received *Helicobacter pylori* eradication therapy seven times. However, despite administration of several antimicrobial agents, her *H. pylori* infection could not be eradicated. We diagnosed her with tubular adenoma in the greater curvature of the gastric antrum at age 61 and IIb‐type diffuse‐type early‐stage gastric cancer in the greater curvature of the gastric upper body at age 71. We performed curative endoscopic submucosal dissection of both tumors.

**FIGURE 1:**
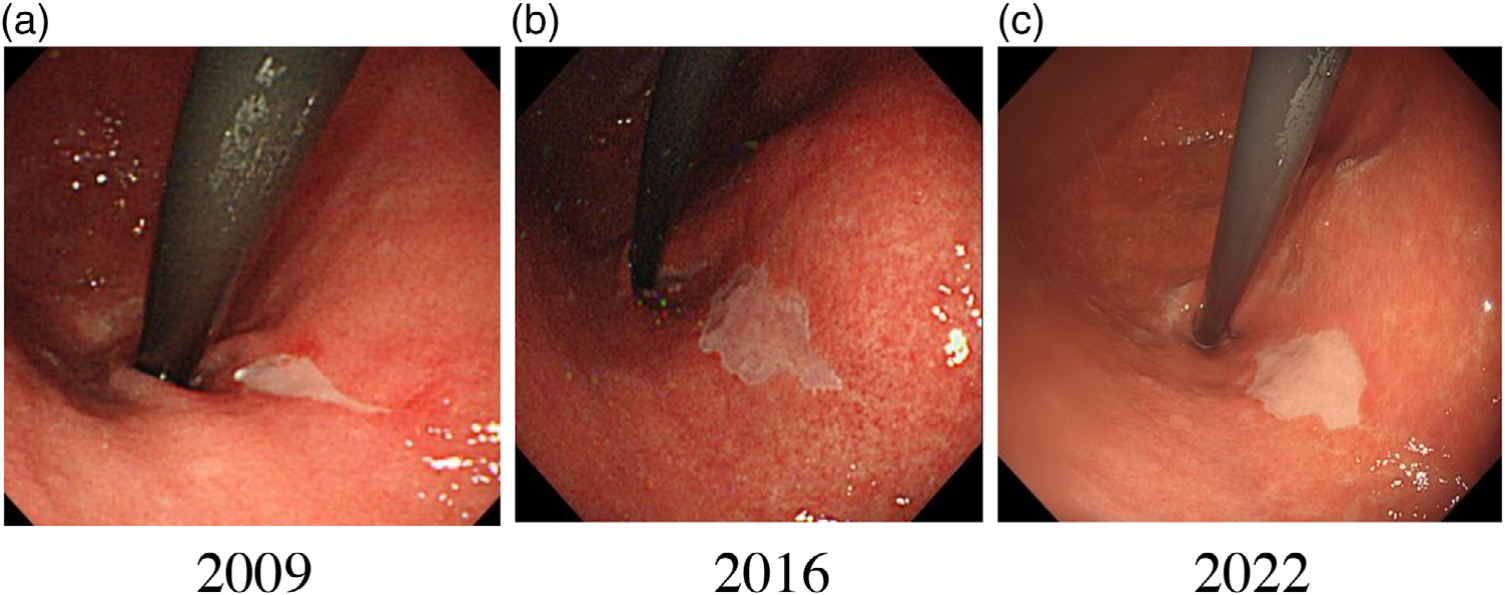
Endoscopic examination of the stomach. A whitish slightly depressed lesion was observed in the lesser curvature of the cardia at the first surveillance in 2009 (10 mm, a) and subsequently in 2016 (15 mm, b) and 2022 (15 mm, c).

Since endoscopy was first performed in 2009 in Tokyo Medical University Hospital, the patient has undergone surveillance endoscopy using ultrathin endoscopy every year for 13 years. In 2009, conventional white light endoscopy (WLI) showed a 10‐mm whitish slightly depressed lesion at the lesser curvature of the gastric cardia (Figure 1a). The gastric mucosa showed open‐type atrophic gastritis with intestinal metaplasia and diffuse redness (Kyoto classification of gastritis: O‐III, A2 IM2 H0 N0 DR2). The physical examination was normal and blood test parameters and tumor markers were within the normal limit. Contrast‐enhanced computed tomography revealed no mass lesions and no lymph node swelling. She routinely took no non‐steroidal anti‐inflammatory drugs, aspirin, steroids, or bisphosphonate drugs.

**FIGURE 2:**
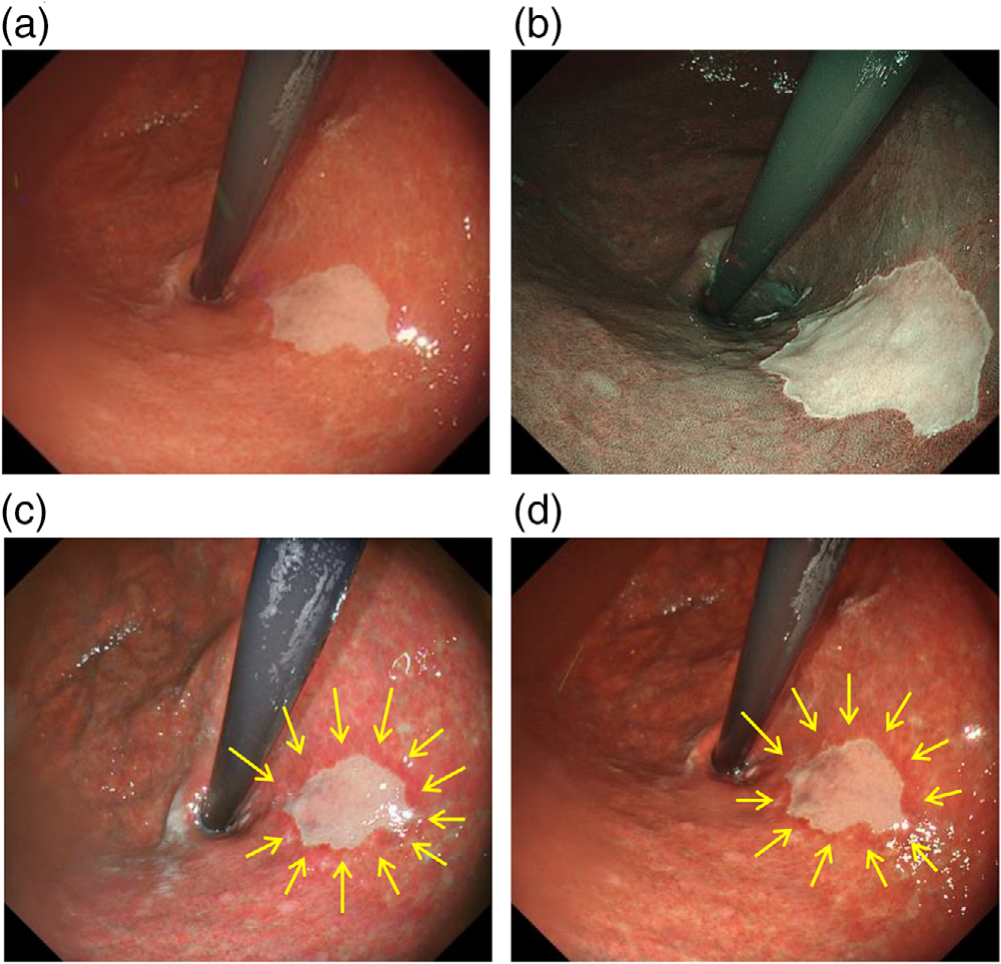
Gastric squamous metaplasia in the gastric cardia. White light imaging (a). Narrow‐band imaging (b) and texture and color enhancement imaging in mode 1 (c) and mode 2 in 2022 (d). Arrows showed the borderline between the lesion and around gastric mucosa and gastric mucosa in both texture and color enhancement imaging modes 1 and 2 were enhanced as reddish changes compared with white light imaging.

**FIGURE 3:**
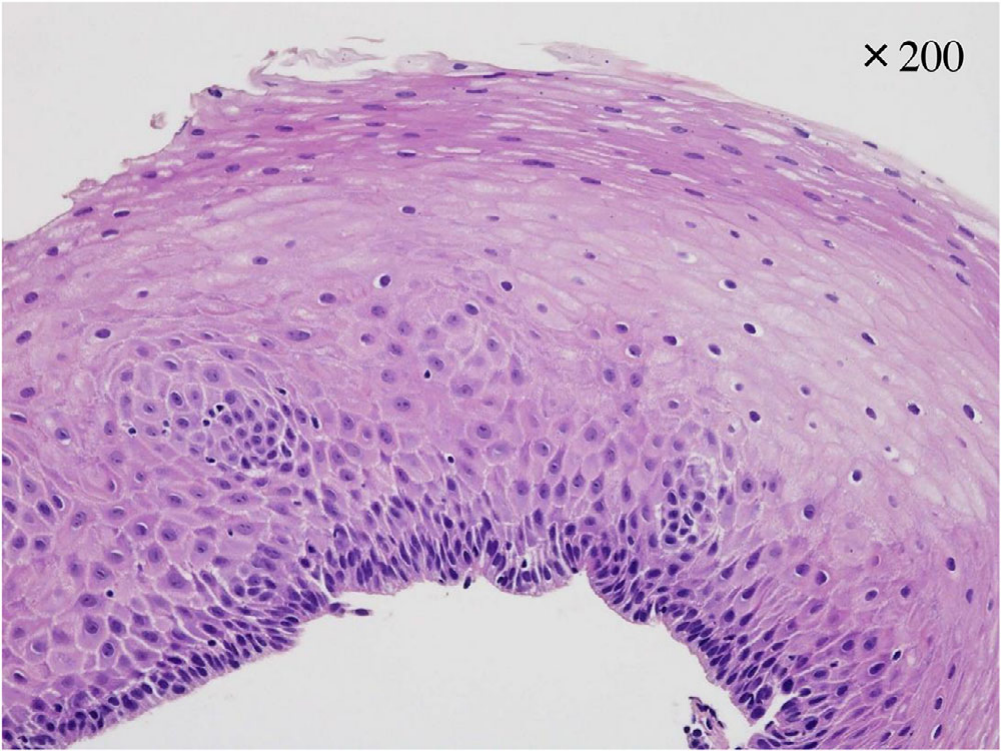
Histological section of a biopsy specimen taken from the gastric whitish lesion. Pathological evaluation revealed stratified squamous epithelial mucosa without infiltration of inflammatory cells or atypia, and no evidence of malignancy (hematoxylin–eosin, magnification: x200).

The lesion gradually increased in size to 15 mm over the 13‐year observation period (Figures 1 and 2a). In the subsequent years after 2009, indigo carmine chromoendoscopy revealed a depressed lesion. Endoscopy with NBI showed a depressed whitish mucosa compared with gastric mucosa with atrophic changes and intestinal metaplasia and dark‐green‐colored esophageal mucosa in the esophagus (Figure 2b). Gastric mucosa‐like structures were observed under whitish esophageal mucosa. TXI in both mode 1 and mode 2 clearly emphasized the presence of a depressed whitish mucosa around the gastric mucosa (Figure 2c,d). Gastric mucosa around the depressed lesion in TXI mode 1 appeared reddish than that in TXI mode 2. IEE using NBI and TXI in mode 1 and mode 2 did not show any abnormality in vascular or structural pattern, and there were no findings suggestive of neoplastic lesions. The biopsy specimen taken from the depressed lesion was pathologically identified as gastric squamous metaplasia without atypia and revealed no evidence of malignancy (Figure 3). We are following up with the patient without endoscopic treatment.

## DISCUSSION

Here, we report an *H. pylori*‐positive patient with gastric squamous metaplasia in gastric cardia monitored using surveillance endoscopy for 13 years, in whom gastric squamous metaplasia was first evaluated by a combination of NBI and TXI.

According to previous studies, gastric squamous metaplasia appears as white or pale mucosal areas mainly located in the gastric cardia and antrum, with lesions that are slightly depressed when sprayed with indigo carmine (Table 1).^1^, ^2^, ^3^, ^4^ In addition to these findings, the present case's metaplasia was not pathologically continuous with the squamous epithelium of the esophagus. Instead, gastric mucosa was observed between the metaplasia and the esophageal squamous epithelium of the esophagus. Although gastric squamous metaplasia has previously been reported in patients with peptic ulcer, tuberculosis, syphilis, corrosive gastritis, pernicious anemia, and aberrant pancreas, the detailed mechanism of how gastric squamous metaplasia develops is unclear. Squamous metaplasia located in other organs such as the lower respiratory tract, bladder, and uterus has also been observed in patients with chronic inflammation and mechanical irritation.^1^ Therefore, the development of gastric squamous metaplasia may be related to long‐term gastric mucosal inflammation induced by *H. pylori* infection, medication, and radiation. In fact, an animal model showed that gastric squamous metaplasia can develop following inflammation induced by injection of pyrogallic acid and methylcholanthrene.^3^ In the present case, chronic inflammation due to persistent *H. pylori* may be responsible for the development and increase in the size of metaplasia.

It is important to endoscopically differentiate between gastric squamous metaplasia, gastric cancer, and peptic ulcer. In addition, there was no report to develop gastric squamous carcinoma from gastric squamous metaplasia after long‐term observation because this metaplasia is often identified together with gastric squamous carcinoma (three case reports of 13 patients for gastric squamous cell carcinoma (SCC), 23.1%), patients with endoscopically‐ and pathologically‐diagnosed gastric squamous metaplasia may be better being carefully followed up for long periods.^7^ However, although correct evaluation of both gastric tumor and gastric squamous metaplasia is important for treatment and surveillance of the lesion, endoscopic evaluation using WLI alone may be difficult and highly dependent on the expertise of the endoscopist. Surveillance endoscopy using NBI is increasingly being considered an effective method for evaluating esophageal lesions, esophageal SCC, and Barrett's esophagus related to esophageal adenocarcinoma worldwide. In addition, NBI revealed a clear border between the esophageal mucosa and gastric mucosa due to the color difference observable with NBI and the ability to observe intrapapillary capillary loops at high magnification in a case of gastric squamous metaplasia.^8^ Observation of intrapapillary capillary loops in gastric squamous metaplasia by endoscopy using NBI may be useful for the diagnosis of this lesion and for discriminating it from esophageal SCC. There has been no report on the observation of gastric squamous metaplasia with TXI modes 1 and 2 (Table 1). However, TXI mode 1 significantly enhances color changes of esophageal SCC and improves the visibility of SCC‐suspicious lesions in the pharynx and esophagus compared with evaluation by WLI (Figure S1).^9^ Therefore, TXI may have the possibility to assist in the endoscopic differentiation between gastric squamous metaplasia and gastric squamous carcinoma. In addition, although only one study examined the usefulness of TXI mode for detecting esophageal SCC compared with NBI, there were no significant differences in color deference and visibility score for the detection of SCC between TXI and NBI.^9^, ^10^ At present, evidence concerning the efficacy of TXI for the detection of SCC is preliminary. Because NBI is currently the standard technique for detecting SCC and esophageal diseases, further studies are needed to evaluate the various IEE modalities for detecting gastrointestinal diseases including gastric squamous metaplasia and gastric SCC.

In conclusion, we described the first report of gastric squamous metaplasia detected using TXI and were able to show that the size of metaplasia gradually changed over time. In previous reports, no cases were endoscopically observable for more than 10 years, and none were observed with TXI mode using a high‐vision ultrathin endoscope. Although gastric squamous metaplasia boundaries in the TXI may have no advantage to observe in the NBI, we propose that the enhancement of lesion structures by NBI and TXI may improve the efficacy of endoscopic diagnosis of gastric squamous metaplasia compared with WLI. When a patient is found to have gastric squamous metaplasia at a health check‐up, we recommend performing long‐term surveillance by WLI and IEE, especially in magnifying NBI, to distinguish it from SCC.

## CONFLICT OF INTEREST STATEMENT

The authors declare no conflict of interest.

## Supporting information


**Supplemental Figure 1**. A case of esophageal squamous cell carcinoma (ESCC). ESCC observed by (A) white‐light imaging, (B) texture and color enhancement imaging mode 1, and (C) mode 2 1.
