# Supplementary material for: Gastric squamous metaplasia observed by image‐enhanced endoscopy
Source: DEN Open. 2023 Mar 14;3(1):e219. doi: 10.1002/deo2.219 (PMC10013409; doi:10.1002/deo2.219)
Supplement: Supplementary file 1 — Supplemental Figure 1. A case of esophageal squamous cell carcinoma (ESCC). ESCC observed by (A) white‐light imaging, (B) texture and color enhancement imaging mode 1, and (C) mode 2 1. [file DEO2-3-e219-s001.zip › deo2219-sup-0001-FigureS1.docx]

**Supplemental Figure legends**

**Supplemental Figure 1**. A case of esophageal squamous cell carcinoma (ESCC). ESCC observed by (A) white-light imaging, (B) texture and color enhancement imaging mode 1, and (C) mode 2 ^1^.

**References**

1 Sugimoto M, Koyama Y, Itoi T, Kawai T. Using texture and colour enhancement imaging to evaluate gastrointestinal diseases in clinical practice: A review. *Ann Med* 2022; **54**: 3315-32.
