# Supplementary figures and images for: Gastric squamous metaplasia observed by image‐enhanced endoscopy
Source: DEN Open. 2023 Mar 14;3(1):e219. doi: 10.1002/deo2.219 (PMC10013409; doi:10.1002/deo2.219)

## Slide 1
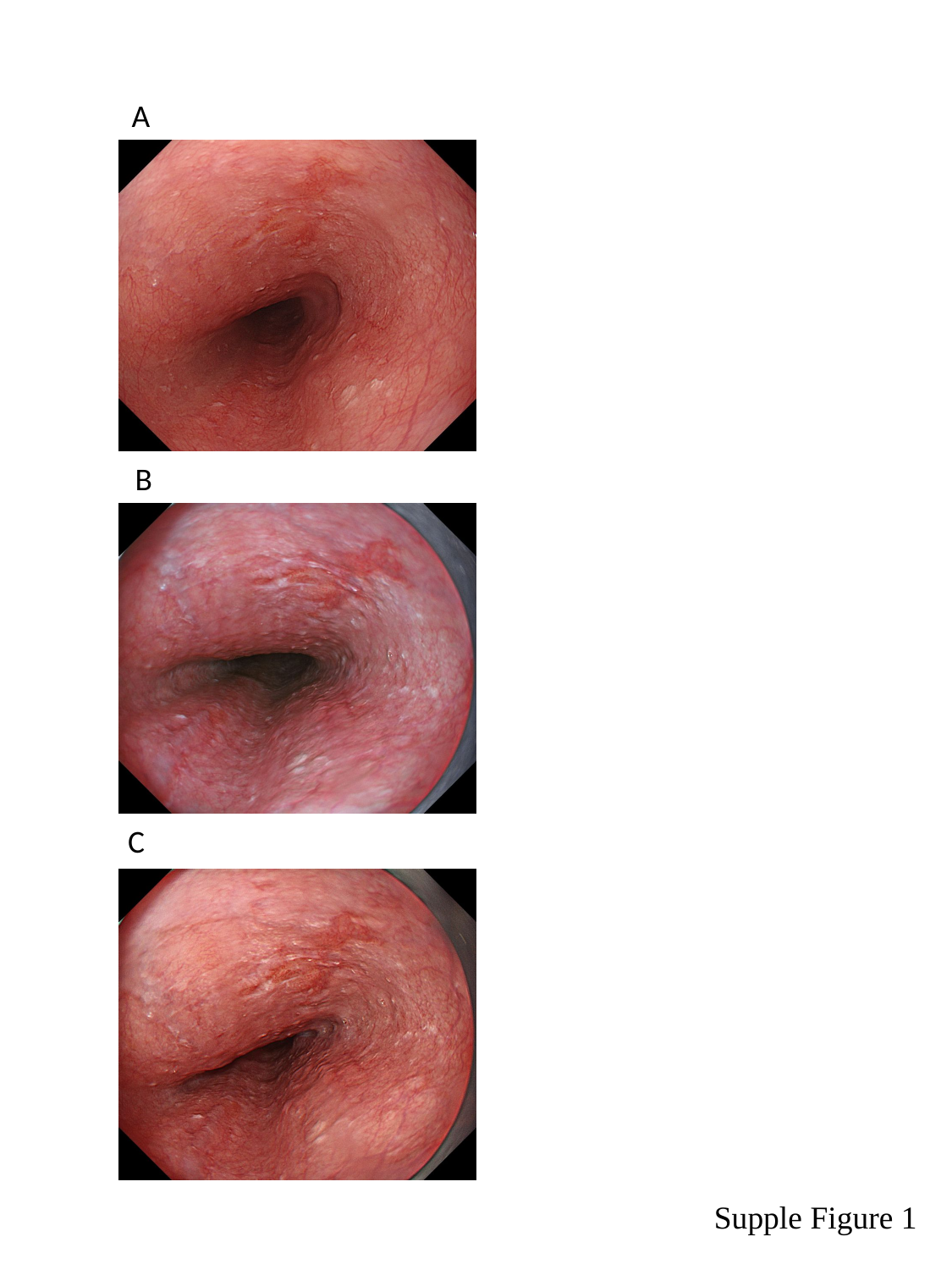

A
B
C
Supple Figure 1

Supplement: Supplementary file 1 — Supplemental Figure 1. A case of esophageal squamous cell carcinoma (ESCC). ESCC observed by (A) white‐light imaging, (B) texture and color enhancement imaging mode 1, and (C) mode 2 1. [file DEO2-3-e219-s001.zip › deo2219-sup-0002-FigureS1.pptx]
